# Association between healthy lifestyle and cognitive decline, all‐cause mortality, and mortality from cardiovascular and cerebrovascular diseases: a 10‐year population‐based prospective cohort study

**DOI:** 10.1002/alz.70021

**Published:** 2025-03-20

**Authors:** Chun Li, Chaobo Bai, Lijun Wang, Mei Zhang, Maigeng Zhou, Jing Chen, Danhua Zhao, Baoyu Chen, Qi Wang, Yuan Li, Junyi Chen, Xintong Guo, Jinjin Wang, Zhe Zhao, Hongqiang Sun, Limin Wang, Junliang Yuan

**Affiliations:** ^1^ National Center for Chronic and Noncommunicable Disease Control and Prevention Chinese Center for Disease Control and Prevention Beijing China; ^2^ Depament of Neurology Peking University Sixth Hospital, Peking University Institute of Mental Health NHC Key Laboratory of Mental Health (Peking University) National Clinical Research Center for Mental Disorders (Peking University Sixth Hospital) Beijing China; ^3^ Department of Pharmacy Peking University Third Hospital Beijing China; ^4^ Institute for Drug Evaluation Peking University Health Science Center Beijing China

**Keywords:** all‐cause mortality, cognitive decline, lifestyle, public health

## Abstract

**INTRODUCTION:**

The association between cognitive function, healthy lifestyle, and mortality remains understudied in large Chinese cohorts.

**METHODS:**

In this nationwide 10‐year prospective study of 24,657 older adults, we assessed Mini‐Mental State Examination (MMSE) categories (<18, 18 to 23, 24 to 27, 28 to 30) and a seven‐component lifestyle score (0 to 7) for their relationships with all‐cause, cardiovascular, and cerebrovascular mortality.

**RESULTS:**

Compared with individuals scoring 28 to 30 on the MMSE, lower scores were linked to elevated all‐cause and cerebrovascular mortality but not cardiovascular mortality. Participants with lifestyle scores of 4 or 5 had a higher risk of all‐cause mortality. Even optimal lifestyle practices did not fully mitigate the heightened mortality risk associated with declining cognitive performance.

**DISCUSSION:**

A healthy lifestyle is beneficial but cannot fully offset the impact of cognitive impairment. Therefore, integrating routine cognitive assessments and targeted interventions with healthy lifestyle practices is crucial for effectively reducing mortality risk.

**Highlights:**

- A nationally representative, 10‐year prospective cohort in China was employed to investigate the combined effects of lifestyle behaviors and cognitive function on all‐cause, cardiovascular, and cerebrovascular mortality.
- Both healthy lifestyle and better cognitive function were associated with a reduced risk of all‐cause mortality.
- Even among individuals practicing optimal lifestyle behaviors, cognitive impairment significantly elevated the risk of all‐cause and cerebrovascular mortality.
- These findings underscore the necessity of incorporating routine cognitive assessments and targeted interventions with healthy lifestyle practices aimed at reducing mortality risk in aging populations.

## 1 INTRODUCTION

Dementia has emerged as a major predictor of mortality globally.^1^, ^2^ According to *The Lancet*, the number of individuals affected by dementia is projected to reach 152 million by 2050, placing immense pressure on global healthcare systems and social support networks.^3^ Age‐related cognitive decline is a well‐established risk factor for dementia and is consistently associated with increased mortality. Recent evidence underscores the importance of healthy lifestyle modifications – such as a balanced diet, regular physical activity, and avoiding smoking and excessive alcohol consumption – in reducing dementia risk and overall mortality.^4^, ^5^, ^6^, ^7^ For example, regular physical activity may boost cerebral blood flow and stimulate the release of neurotrophic factors, while diets rich in antioxidants and essential nutrients help reduce oxidative stress and maintain neuronal integrity. By supporting synaptic plasticity and vascular integrity, these lifestyle factors can slow cognitive decline and, in turn, potentially lower long‐term mortality risk.^8^, ^9^, ^10^, ^11^ Therefore, lifestyle interventions not only reduce the risk of cardiovascular diseases but also play a crucial role in preserving cognitive function and promoting longevity.^8^, ^12^, ^13^, ^14^, ^15^

In China, dementia currently affects an estimated 15.07 million individuals aged 60 and older (6.0%), while an additional 38.77 million (15.5%) experience mild cognitive impairment. The rising incidence of cognitive disorders represents a significant public health challenge, particularly as the elderly population continues to grow.^16^ For instance, a longitudinal study including 1,996 older adults in Beijing found that cognitive decline, measured using the Mini‐Mental State Examination (MMSE), was strongly associated with increased risks of cardiovascular and all‐cause mortality.^17^ Similarly, data from the Chinese longitudinal healthy longevity survey, which included 5124 participants aged 80 and older, revealed that cognitive decline significantly increased the risk of all‐cause mortality.^18^ Notably, the study also highlighted that adopting healthier lifestyle habits, especially improving dietary choices, notably reduced mortality risk. Other studies further emphasize the critical interaction between cognitive function and lifestyle factors in influencing overall mortality risk among aging populations.^19^, ^20^, ^21^

Although previous studies independently assessed the associations between lifestyle factors, cognitive function, and mortality, comprehensive investigations into their complex interrelationships and collective impact on mortality risk remain limited.^19^, ^22^ Notably, there is a lack of nationally representative, long‐term cohort studies exploring these dynamics within China. To our knowledge, this study is the first nationwide, prospective cohort analysis based on the China Chronic Disease and Risk Factor Surveillance (CCDRFS), involving over 20,000 participants with a 10‐year follow‐up period. The primary aim of this research was to comprehensively explore the relationships between lifestyle, cognitive function, and the risks of all‐cause, cardiovascular, and cerebrovascular mortality. This study sought to address critical gaps in the existing literature and provide valuable evidence to inform public health interventions.

## 2 METHODS

### 2.1 Study design and participants

This study utilized data from the 2010 to 2011 CCDRFS as the baseline survey, linking individual records to the National Mortality Surveillance System (NMSS) to establish a 10‐year prospective cohort (Figures S1‐1, S1‐2). The CCDRFS is a nationwide disease surveillance survey conducted by the National Center for Chronic and Noncommunicable Disease Control and Prevention. Detailed descriptions of its design and methodology are available elsewhere.^23^ Briefly, the 2010 CCDRFS covered 162 disease surveillance points (DSPs) across all 31 provinces, autonomous regions, and municipalities in China, ensuring representation of both urban and rural populations. A multistage stratified cluster random sampling approach was employed to recruit participants. During the survey, trained investigators collected demographic data, conducted face‐to‐face interviews, administered structured questionnaires, and performed physical examinations. Blood and urine samples were also collected, along with on‐site testing for hemoglobin, glucose, and lipids. Rigorous quality control measures were implemented to ensure the reliability and consistency of data collection. The CCDRFS received ethical approval from the Institutional Review Board of the China Centers for Disease Control and Prevention (Approval No. 201010), and all participants provided written informed consent prior to participation. The NMSS is a comprehensive mortality surveillance system that records nationwide death records from over 600 DSPs, covering approximately 24.3% of the national population. Death records, including date, status, and cause of death (coded using the International Classification of Diseases, 10th Revision [ICD‐10]), were linked to CCDRFS data using personal identifiers such as ID numbers, demographic information, and residential details. This linkage enabled accurate ascertainment of mortality outcomes for over 90% of the participants. Further details on the NMSS can be found in other sources.^24^

### 2.2 Outcome measures

The primary outcomes of this study included all‐cause mortality, cardiovascular mortality, and cerebrovascular mortality. Mortality data were sourced from the NMSS and carefully linked to baseline records from CCDRFS using unique personal identifiers such as ID numbers, demographic characteristics, and residential information, ensuring a high degree of accuracy in data integration.

**RESEARCH IN CONTEXT**

1. **Systematic review**: The authors reviewed the literature using PubMed sources examining longitudinal research on how lifestyle factors and cognitive function influence mortality. Studies have shown that healthier lifestyles and better cognition can reduce premature death risk. However, data on their combined effects, particularly in large, nationally representative Chinese cohorts, remain insufficient.
2. **Interpretation**: We observed that a healthier lifestyle lowered all‐cause mortality, yet cognitive decline was a significant contributor to mortality risk. Even among individuals practicing optimal lifestyle behaviors, lower MMSE scores markedly elevated both all‐cause and cerebrovascular mortality risks, highlighting the essential interplay between lifestyle and cognition.
3. **Future directions**: Additional longitudinal studies are needed to clarify how evolving lifestyle patterns and cognitive trajectories interact. Integrating advanced measurement methods (eg, wearable devices, comprehensive dietary indices, biomarker assessments) and prospective trials combining cognitive training with personalized lifestyle interventions could further identify effective strategies for reducing mortality.

### 2.3 Assessment of cognitive function and lifestyle factors

Cognitive function was assessed using the MMSE, a widely recognized standardized tool for cognitive screening. Participants who indicated potential memory issues through affirmative responses to memory‐related screening questions were required to complete the MMSE. Baseline MMSE scores were stratified into four categories: <18, 18 to 23, 24 to 27, and 28 to 30 (reference group). A score of ≥28 is commonly considered indicative of normal cognitive function, while thresholds of <24 and <18 are used to identify individuals with suspected mild cognitive impairment and severe cognitive impairment, respectively.^17^, ^25^, ^26^

Lifestyle factors were assessed at baseline using a structured questionnaire, covering smoking status (categorized as “current smoker,” “past smoker,” or “never smoked”), alcohol consumption (classified as “never,” “previous,” or “current”), and physical activity, measured in metabolic equivalents (METs) and categorized as “low,” “moderate,” or “high.” Dietary habits were evaluated based on adherence to dietary guidelines, including daily intake of fruits, vegetables, and red meat. Sleep duration was classified into three categories: less than 7 h, 7 to 8 h, and more than 8 h per day. Sedentary time, measured in hours per day, was categorized as less than 4 h or 4 h and above. Living arrangements were classified as either living with others or living alone. A composite lifestyle score was derived by summing individual component scores, with higher totals indicating more health‐promoting behaviors. Based on these scores, participants were divided into three lifestyle categories: 0 to 3, 4 or 5, and 6 or 7^27^ (Table S1).

### 2.4 Covariates

The analysis considered a wide range of covariates, including age, sex, education level (categorized as less than college and college/university degree or higher), marital status (married vs unmarried/divorced/widowed), ethnicity (Han vs other ethnicities), residence type (urban vs rural), body mass index (BMI), income (annual household income <10,000 yuan, 10,000 to 30,000 yuan, and >30,000 yuan), and employment status (employed vs unemployed). The analysis also accounted for preexisting conditions, such as hypertension, diabetes mellitus, hyperlipidemia, myocardial infarction, cerebrovascular disease, chronic obstructive pulmonary disease (COPD), cancer, traumatic brain injury, and depression.

### 2.5 Statistical analyses

Descriptive statistics were used to summarize the baseline characteristics of participants across lifestyle score categories. Continuous variables were presented as means with standard deviations (SD), while categorical variables were expressed as frequencies and percentages. Cox proportional hazards regression models were employed to assess the associations between baseline MMSE scores, lifestyle factors, and the risks of all‐cause mortality, cardiovascular disease mortality, and cerebrovascular disease mortality. Hazard ratios (HRs) with 95% confidence intervals (CIs) were calculated for each factor. Three sequential models were constructed: Model 1 adjusted for age and sex; Model 2 additionally adjusted for age, sex, education level, marital status, ethnicity, residence, BMI, income, and occupation; and Model 3 adjusted for age, sex, education level, marital status, ethnicity, residence, BMI, income, occupation, hypertension, diabetes mellitus, hyperlipidemia, myocardial infarction, cerebrovascular disease, COPD, cancer, traumatic brain injury, and depression. Lifestyle factors were analyzed both individually (eg, smoking, alcohol consumption, physical activity, diet, sleep duration, sedentary time, and living arrangements) and as a composite lifestyle score categorized into three groups (0 to 3, 4 to 5, 6 to 7). Interaction terms were tested to explore whether the lifestyle score modified the effect of MMSE categories on the outcomes. All statistical tests were two‐sided, with a *p* value < 0.05 considered statistically significant. Analyses were conducted using SAS (version 9.4; SAS Institute, Cary, NC).

## 3 RESULTS

A total of 24,657 participants from the CCDRFS were categorized into three lifestyle score groups: low score (0 to 3 points, 4.32%), moderate score (4 to 5 points, 41.85%), and high score (6 to 7 points, 53.83%) (Table 1). The study included a 10‐year follow‐up period, with participants having a mean age of 60.85 ± 8.55 years and similar age distributions across the groups. Gender distribution analysis revealed a significantly higher proportion of females in the high‐score group compared to the low‐score group. The low‐score group was characterized by a higher proportion of individuals with a college degree or higher, urban residency, and marital status than the moderate and high‐score groups. Conversely, the low‐score group also had a greater percentage of participants with an annual income below 10,000 yuan and a higher prevalence of preexisting conditions such as COPD, depression, and shorter sleep duration. Cognitive function was assessed using the MMSE. The results indicated that the low‐score group had fewer participants scoring below 18, a smaller proportion in the range of 18 to 23, and a greater percentage scoring above 24.

**TABLE 1:** Baseline characteristics of CCDRFS participants by lifestyle score category.

	Lifestyle score		
Demographic characteristics	0 to 3(*n* = 1066)	4 to 5(*n* = 10318)	6 to 7(*n* = 13,273)
Female, *n* (%)	104 (9.76)	3334 (32.31)	9424 (71.00)
Age (mean; SD), years	61.32 (9.42)	61.35 (8.88)	60.45 (8.19)
College/university degree, *n* (%)	256 (24.02)	2002 (19.40)	1867 (14.07)
Han ethnicity, *n* (%)	993 (93.15)	9382 (90.93)	11,758 (88.59)
Married, *n* (%)	777 (72.89)	8451 (81.91)	11,385 (85.78)
Urban, *n* (%)	500 (46.90)	4398 (42.62)	5293 (39.88)
BMI (mean; SD) (kg/m^2^)	23.77 (3.38)	23.86 (3.46)	24.42 (3.59)
Annual household income, *n* (%)			
<10,000 RMB	284 (26.64)	2456 (23.80)	2709 (20.41)
10,000 to <30,000 RMB	373 (34.99)	3759 (36.43)	5268 (39.69)
≥30000 RMB	409 (38.37)	4103 (39.77)	5296 (39.90)
Employed, *n* (%)	732 (68.67)	6720 (65.13)	7755 (58.43)
Previous diseases			
Hypertension, *n* (%)	625 (58.63)	5841 (56.61)	7823 (58.94)
Diabetes mellitus, *n* (%)	179 (16.79)	1652 (16.01)	2215 (16.69)
Myocardial infarct, *n* (%)	10 (0.94)	112 (1.09)	138 (1.04)
Cerebrovascular disease, *n* (%)	17 (1.59)	144 (1.40)	138 (1.04)
Hyperlipidemia, *n* (%)	558 (52.35)	5528 (53.58)	7243 (54.57)
COPD, *n* (%)	71 (6.66)	623 (6.04)	634 (4.78)
Cancer, *n* (%)	3 (0.28)	52 (0.50)	69 (0.52)
Traumatic brain injury, *n* (%)	5 (0.47)	46 (0.45)	68 (0.51)
Depression, *n* (%)	26 (2.44)	223 (2.16)	218 (1.64)
Living alone, *n* (%)	235 (22.05)	2316 (22.45)	3117 (23.48)
Sleep duration (mean; SD), hours/day	7.00 (1.94)	7.35 (1.68)	7.49 (1.25)
MMSE score*			
MMSE < 18	29 (8.63)	342 (11.26)	474 (11.44)
MMSE 18 to 23	60 (17.86)	622 (20.47)	1061 (25.60)
MMSE 24 to 27	99 (29.46)	819 (26.96)	1109 (26.76)
MMSE 28 to 30	148 (44.05)	1255 (41.31)	1500 (36.20)

*Note*: The CCDRFS required participants to answer three questions: (1) Compared to 12 months ago, has your memory worsened? (2) Do you feel that your memory is worse than that of relatives, neighbors, or colleagues of the same age? (3) Do your family members, neighbors, or colleagues think that your memory is poor? If you answered yes to any of these three questions, the participant proceeded to complete the MMSE questionnaire. Ultimately, a total of 7407 individuals completed the MMSE questionnaire. Thus, valid data were utilized for all variables.

Abbreviations: BMI, body mass index; CCDRFS, China Chronic Disease and Risk Factor Surveillance; COPD, chronic obstructive pulmonary disease; MMSE, Mini‐Mental State Examination; RMB, Renminbi; SD, standard deviation.

Over the 10‐year follow‐up, 3028 participants (12.28% of the total cohort) died from all causes, including 1272 deaths (5.16%) attributed to cardiovascular and cerebrovascular diseases. Analysis revealed that specific lifestyle factors were significantly associated with all‐cause, cardiovascular, and cerebrovascular mortality risks (Table 2). Current smokers had a substantially higher risk of all‐cause mortality compared to former or never smokers (HR = 1.29, 95% CI: 1.17 to 1.41, *p *< 0.001). Similarly, participants with insufficient physical activity exhibited an increased risk of both all‐cause mortality (HR = 1.12, 95% CI: 1.02 to 1.22, *p* < 0.05) and cardiovascular mortality (HR = 1.30, 95% CI: 1.07 to 1.59, *p* < 0.001) (Table S2). Individuals with a daily sleep duration exceeding 8 h demonstrated significantly higher risks for all‐cause mortality (HR = 1.20, 95% CI: 1.10 to 1.31, *p* < 0.001) and cerebrovascular mortality (HR = 1.23, 95% CI: 1.03 to 1.48, *p* < 0.05). Conversely, participants with a daily sedentary time of ≥4 h showed a reduced risk of cerebrovascular mortality (HR = 0.72, 95% CI: 0.56 to 0.93, *p* < 0.05) (Table S3). Moreover, the overall lifestyle score was significantly associated with all‐cause mortality risk. Compared to individuals with scores of 6 to 7, those with scores of 4 to 5 had a notably higher risk of all‐cause mortality (HR = 1.14, 95% CI: 1.05 to 1.23, *p* < 0.001) (Table 2). A progressive decline in MMSE scores was strongly associated with an increased risk of all‐cause mortality, with HR of 1.19, 1.34, and 1.49 for scores of 24 to 27, 18 to 23, and below 18, respectively, compared to the reference group (28 to 30). Similarly, cerebrovascular disease mortality followed a comparable trend, with risks increasing by 43% (95% CI: 0.99 to 2.07, *p *= 0.05), 71% (95% CI: 1.18 to 2.50, *p* < 0.001), and 115% (95% CI: 1.41 to 3.26, *p* < 0.001) as MMSE scores declined. In contrast, no significant associations were observed between reductions in MMSE score and cardiovascular mortality (Table 3).

**TABLE 2:** Hazard ratio (95% CI) of all‐cause mortality based on individual and combined lifestyle factors.

	No. cases/100,000 person‐years	HR (95% CI)		
		Model 1	Model 2	Model 3
Smoking				
Previous/never	869.86	1 (Reference)	1 (Reference)	1 (Reference)
Current	1391.57	1.33 (1.21 to 1.46)^*^	1.28 (1.16 to 1.40)^*^	1.29 (1.17 to 1.41)^*^
Alcohol consumption				
Never	1054.67	1 (Reference)	1 (Reference)	1 (Reference)
Previous/current	1071.65	0.94 (0.87 to 1.03)	0.96 (0.89 to 1.04)	0.97 (0.89 to 1.05)
Physical activity				
Adequate	983.27	1 (Reference)	1 (Reference)	1 (Reference)
Inadequate	1490.29	1.13 (1.04 to 1.24)^*^	1.13 (1.03 to 1.23)^*^	1.12 (1.02 to 1.22)^**^
Healthy diet				
Yes	1053.43	1 (Reference)	1 (Reference)	1 (Reference)
No	1367.97	1.18 (0.95 to 1.46)	1.08 (0.87 to 1.34)	1.03 (0.83 to 1.28)
Sleep duration, h/day				
7 to 8	928.64	1 (Reference)	1 (Reference)	1 (Reference)
≤6	1151.05	1.08 (1.00 to 1.18)	1.06 (0.97 to 1.15)	1.04 (0.95 to 1.13)
≥9	1321.66	1.26 (1.15 to 1.38)^*^	1.22 (1.11 to 1.33)^*^	1.20 (1.10 to 1.31)^*^
Sedentary time, h/day				
<4	1086.29	1 (Reference)	1 (Reference)	1 (Reference)
≥4	927.02	0.87 (0.79 to 0.96)^*^	0.97 (0.87 to 1.08)	0.97 (0.87 to 1.08)
Living alone				
No	999.57	1 (Reference)	1 (Reference)	1 (Reference)
Yes	1878.21	1.19 (1.06 to 1.33)^*^	0.99 (0.87 to 1.12)	0.99 (0.87 to 1.12)
Overall lifestyle score				
6 to 7	870.71	1 (Reference)	1 (Reference)	1 (Reference)
4 to 5	1257.30	1.16 (1.07 to 1.25)^*^	1.13 (1.04 to 1.22)^*^	1.14 (1.05 to 1.23)^*^
0 to 3	1558.62	1.22 (1.04 to 1.42)^**^	1.14 (0.97 to 1.33)	1.13 (0.96 to 1.32)

*Note*: Model 1 adjusted for age, sex; Model 2 adjusted for age, sex, education level, marital status, ethnicity, residence, BMI, income, occupation; Model 3 adjusted for age, sex, education level, marital status, ethnicity, residence, BMI, income, occupation, hypertension, diabetes mellitus, hyperlipidemia, myocardial infarction, cerebrovascular disease, COPD, cancer, traumatic brain injury, depression.

Abbreviations: CI, confidence interval; COPD, chronic obstructive pulmonary disease; HR, hazard ratio.

* *p *< 0.001;

** *p *< 0.05.

**TABLE 3:** Hazard ratio (95% CI) for all‐cause, cardiovascular disease and cerebrovascular disease mortality according to baseline MMSE score.

	No. cases/100,000 person‐years	Model 1	Model 2	Model 3
		HR (95% CI)	HR (95% CI)	HR (95% CI)
**All‐cause mortality**				
MMSE < 18	2145.79	2.38 (1.99 to 2.84)^*^	1.54 (1.27 to 1.86)^*^	1.49 (1.21 to 1.84)^*^
MMSE 18 to 23	1498.30	1.64 (1.40 to 1.93)^*^	1.36 (1.15 to 1.60)^*^	1.34 (1.12 to 1.60)^*^
MMSE 24 to 27	1286.35	1.41 (1.20 to 1.65)^*^	1.25 (1.06 to 1.46)^**^	1.19 (1.01 to 1.41)^**^
MMSE 28 to 30	919.15	1 (Reference)	1 (Reference)	1 (Reference)
**Cardiovascular disease mortality**				
MMSE < 18	407.70	2.34 (1.55 to 3.51)^*^	1.09 (0.71 to 1.68)	0.95 (0.59 to 1.54)
MMSE 18 to 23	305.77	1.72 (1.20 to 2.47)^*^	1.19 (0.83 to 1.73)	1.12 (0.75 to 1.66)
MMSE 24 to 27	201.27	1.13 (0.77 to 1.66)	0.92 (0.62 to 1.36)	0.84 (0.56 to 1.26)
MMSE 28 to 30	178.39	1 (Reference)	1 (Reference)	1 (Reference)
**Cerebrovascular disease mortality**				
MMSE < 18	697.38	4.29 (3.00 to 6.15)^*^	2.72 (1.86 to 3.98)^*^	2.15 (1.41 to 3.26)^*^
MMSE 18 to 23	417.89	2.54 (1.80 to 3.57)^*^	2.05 (1.45 to 2.91)^*^	1.71 (1.18 to 2.50)^*^
MMSE 24 to 27	310.65	1.87 (1.32 to 2.66)^*^	1.64 (1.15 to 2.34)^*^	1.43 (0.99 to 2.07)
MMSE 28 to 30	166.29	1 (Reference)	1 (Reference)	1 (Reference)

*Note*: Model 1 adjusted for age, sex; Model 2 adjusted for age, sex, education level, marital status, ethnicity, residence, BMI, income, occupation; Model 3 adjusted for age, sex, education level, marital status, ethnicity, residence, BMI, income, occupation, hypertension, diabetes mellitus, hyperlipidemia, myocardial infarction, cerebrovascular disease, COPD, cancer, traumatic brain injury, depression.

Abbreviations: COPD, chronic obstructive pulmonary disease; CI, confidence interval; HR, hazard ratio; MMSE, Mini‐Mental State Examination.

* *p *< 0.001;

** *p *< 0.05.

Age‐stratified analysis among participants under 65 revealed a significant increase in all‐cause mortality with declining MMSE scores, with risks rising by 30% (95% CI: 1.02 to 1.66, *p *< 0.05) at moderate score reductions, 52% (95% CI: 1.16 to 2.00, *p *< 0.05) at greater reductions, and 62% (95% CI: 1.10 to 2.39, *p *< 0.05) at the lowest scores (Table S4). Gender‐stratified analysis indicated that both males and females experienced an increased risk of all‐cause mortality with declining MMSE scores. However, the rise in cerebrovascular mortality risk was notably more pronounced among males. However, the rise in cerebrovascular mortality risk was notably more pronounced in men. Among men, each five‐point reduction in MMSE scores was associated with increased risks of 56% (95% CI: 1.00 to 2.44, *p *< 0.05), 79% (95% CI: 1.12 to 2.88, *p *< 0.05), and 80% (95% CI: 1.02 to 3.19, *p *< 0.05) as scores progressively declined (Table S5).

A significant association was observed between lower baseline MMSE scores and an increased risk of all‐cause mortality, particularly in participants with higher lifestyle scores (6 to 7). This association remained robust even after fully adjusting for covariates. Compared to individuals with MMSE scores of 28 to 30, each five‐point decline in MMSE score corresponded to a 35% increase in all‐cause mortality risk (95% CI: 1.05 to 1.74, *p *< 0.05), a 49% increase (95% CI: 1.15 to 1.93, *p *< 0.001), and a 74% increase (95% CI: 1.28 to 2.36, *p *< 0.001) at the lowest MMSE score levels (Table 4).

**TABLE 4:** Association of baseline MMSE scores with all‐cause mortality: stratification by lifestyle factors.

	HR (95%*CI*)		
	Model 1	Model 2	Model 3
All‐cause mortality Lifestyle score 0 to 3			
MMSE < 18	2.66 (1.19 to 5.92)^**^	1.46 (0.61 to 3.49)	1.10 (0.41 to 2.92)
MMSE 18 to 23	2.69 (1.39 to 5.22)^*^	2.01 (1.01 to 4.00)^**^	1.28 (0.56 to 2.90)
MMSE 24 to 27	1.41 (0.73 to 2.74)	1.11 (0.56 to 2.19)	0.80 (0.37 to 1.72)
MMSE 28 to 30	1 (Reference)	1 (Reference)	1 (Reference)
Lifestyle score 4 to 5			
MMSE < 18	2.38(1.86 to 3.05)^*^	1.50(1.15 to 1.96)^*^	1.33(1.00 to 1.78)
MMSE 18 to 23	1.64(1.31 to 2.06)^*^	1.28(1.01 to 1.62)^**^	1.15(0.89 to 1.48)
MMSE 24 to 27	1.36(1.09 to 1.69)^*^	1.20(0.96 to 1.50)	1.13(0.89 to 1.42)
MMSE 28 to 30	1 (Reference)	1 (Reference)	1 (Reference)
Lifestyle score 6 to 7			
MMSE < 18	2.44 (1.85 to 3.20)^*^	1.65 (1.24 to 2.19)^*^	1.74 (1.28 to 2.36)^*^
MMSE 18 to 23	1.77 (1.39 to 2.24)^*^	1.46 (1.15 to 1.86)^*^	1.49 (1.15 to 1.93)^*^
MMSE 24 to 27	1.52 (1.19 to 1.94)^*^	1.36 (1.06 to 1.73)^**^	1.35 (1.05 to 1.74)^**^
MMSE 28 to 30	1 (Reference)	1 (Reference)	1 (Reference)

*Note*: Model 1 adjusted for age, sex; Model 2 adjusted for age, sex, education level, marital status, ethnicity, residence, BMI, income, occupation; Model 3 adjusted for age, sex, education level, marital status, ethnicity, residence, BMI, income, occupation, hypertension, diabetes mellitus, hyperlipidemia, myocardial infarction, cerebrovascular disease, COPD, cancer, traumatic brain injury, depression.

Abbreviations: COPD, chronic obstructive pulmonary disease; CI, confidence interval; HR, hazard ratio; MMSE, mini‐mental state examination.

* *p *< 0.001;

** *p *< 0.05.

For cerebrovascular mortality, a similar trend of escalating risk was observed with decreasing MMSE scores. Specifically, individuals with MMSE scores of 24 to 27 had a 136% higher risk (95% CI: 1.29 to 4.33, *p *< 0.05), those scoring 18 to 23 showed a 241% increase (95% CI: 1.86 to 6.25, *p *< 0.001), and participants with scores below 18 experienced a 348% elevation in cerebrovascular mortality risk (95% CI: 2.29 to 8.80, *p *< 0.001) (Table S6). In contrast, no significant associations were found between MMSE score declines and cardiovascular mortality (Table S7). In the low lifestyle score group (0 to 3), the HRs for all‐cause mortality across MMSE categories showed wide CIs and lacked statistical significance (eg, MMSE < 18: HR = 1.10, 95% CI: 0.41 to 2.92), likely due to the limited sample size in this subgroup. These findings underscore the complexities of stratified analyses and emphasize the need for cautious interpretation of subgroup results.

## 4 DISCUSSION

Maintaining a healthy lifestyle and preserving cognitive function are crucial in preventing premature mortality.^15^, ^28^, ^29^, ^30^ In our large‐scale, 10‐year prospective cohort study, we observed that both healthy lifestyle and better cognitive function were associated with a reduced risk of all‐cause mortality. Importantly, individuals with lower MMSE scores had a significantly higher risk of all‐cause mortality, even among those with the optimal lifestyle scores (6 to 7 points). This finding suggests that cognitive decline may increase mortality risk, even in the context of a healthy lifestyle. Therefore, while lifestyle interventions are important, integrating cognitive health promotion into comprehensive public health strategies is essential to maximize population survival rates.

In our study, current smoking, insufficient physical activity, and prolonged sleep duration (>8 h/day) were significantly associated with increased all‐cause mortality. These findings are consistent with established evidence linking smoking and inadequate physical activity to elevated mortality risk, highlighting the detrimental impact of these behaviors on overall health.^31^, ^32^ Notably, the association between extended sleep duration and increased mortality supports prior research identifying a U‐shaped relationship between sleep and mortality, where both insufficient and excessive sleep are linked to higher mortality rates.^33^ Participants with lifestyle scores of 4 to 5 demonstrated a significantly higher risk of all‐cause mortality compared to those with higher scores (6 to 7). While participants with the lowest lifestyle scores (0 to 3) showed a tendency toward increased all‐cause mortality, this association did not reach statistical significance after full adjustment. These findings underscore the critical importance of optimizing lifestyle behaviors to reduce mortality risk, particularly by addressing modifiable factors such as smoking, physical activity, and sleep patterns.^18^, ^20^, ^28^

Our findings reveal a significant association between lower MMSE scores and increased risks of both all‐cause and cerebrovascular mortality. Compared to participants with MMSE scores of 28 to 30, those scoring 24 to 27, 18 to 23, and below 18 demonstrated a 19%, 34%, and 49% higher risk of all‐cause mortality, respectively. These findings build on prior research by employing a broader, nationally representative cohort with a wider age range and a larger sample size.^17^, ^18^ Notably, while cognitive impairment was strongly linked with cerebrovascular mortality, no such relationship was observed with cardiovascular mortality in our study. This discrepancy may reflect methodological and population‐specific differences. For instance, O'Donnell et al. focused on individuals with preexisting cardiovascular disease or high‐risk diabetes, both of which significantly elevate cardiovascular mortality risk.^15^ Similarly, the Beijing Longitudinal Study of Aging (BLSA) by An et al. was limited by a relatively small sample size of 1996 participants, with reducing the external validity and generalizability of its findings.^17^ In contrast, our study utilized a nationally representative cohort of 24,657 participants with diverse baseline characteristics, providing a more nuanced and comprehensive perspective on cardiovascular mortality trends within the general population.

Of note, the association between low MMSE scores and mortality was particularly pronounced in participants younger than 65, underscoring the critical importance of early cognitive screening and timely intervention in this age group.^34^ Additionally, gender‐stratified analysis showed no significant difference in the relationship between lower MMSE scores and mortality risk for men and women, suggesting that cognitive decline has a consistent impact on mortality across genders.^7^, ^19^ In our analysis of the combined effects of cognitive function and lifestyle factors on mortality risk, we observed that among participants with high lifestyle scores (6 to 7), lower MMSE scores were significantly associated with increased all‐cause mortality. In particular, within this group, participants with MMSE scores of 24 to 27, 18 to 23, and below 18 faced 35%, 49%, and 74% higher risks of all‐cause mortality, respectively, compared to those with scores of 28 to 30. A similar trend was noted for cerebrovascular mortality, suggesting that vascular‐related cognitive decline may contribute to elevated mortality risk.^35^, ^36^, ^37^, ^38^ Taken together, these findings highlight the intricate interplay between cognitive function and lifestyle behaviors. Regular exercise, a balanced diet, and avoidance of smoking or excessive alcohol can help mitigate the vascular and inflammatory insults often linked to cognitive decline.^14^, ^39^ Meanwhile, individuals with preserved cognition typically exhibit better self‐management – such as consistent medication adherence and more informed health decisions – as well as stronger social engagement and psychological well‐being, thereby reducing risks like depression and social isolation. Although healthy lifestyle practices cannot entirely negate cognitive impairment, they collectively slow its progression and lessen its ultimate impact on all‐cause mortality.

Despite the significant findings of this study, several limitations should be acknowledged. First, the sample size in the low lifestyle score group (0 to 3 points) was relatively small, leading to increased variability in the data and wider CIs. This limited the stability of the association between lifestyle and mortality in this group. Consequently, the results for this group should be interpreted with caution. Future research should aim to increase the sample size, particularly in the low lifestyle‐score group and among specific disease populations, to improve the stability and reliability of these estimates. Second, education level was assessed through self‐report, which did not capture the quality of education or the actual learning experience. Since education quality varies across regions and between urban and rural areas, this limitation might have led to an underestimation of the true effect of education on mortality. Third, mortality data were obtained from the NMSS. While most death records were accurately linked, underreporting remains a potential concern, particularly due to regional variations in reporting quality. However, because our analysis focused on overall all‐cause mortality, rather than regional trends, and underreporting appeared random, the impact on the results is likely minimal. Additionally, the design of the CCDRFS may have underestimated cognitive decline, as only participants reporting memory concerns were prompted to complete the MMSE. Nonetheless, all analyses were conducted using valid data, ensuring the rigor and reliability of the findings. In future studies, we aim to implement repeated assessments of both lifestyle factors and cognitive function to further elucidate their longitudinal interactions with all‐cause mortality. Finally, although we adjusted for a broad range of covariates, some potential confounders and unmeasured residual factors could not be fully accounted for, which may have influenced the results.

## 5 CONCLUSION

While maintaining a healthy lifestyle provides significant benefits, it cannot fully mitigate the impact of cognitive impairment. Cognitive decline can impair an individual's capacity to engage in health‐promoting behaviors, adhere to medical treatments, and recognize health risks, ultimately increasing mortality risk. Our findings highlight the critical importance of prioritizing cognitive health alongside the promotion of healthy lifestyle behaviors to reduce mortality risk. Public health strategies should extend their scope beyond advocating for healthy habits to include routine cognitive assessments and targeted interventions aimed at preventing cognitive decline. By integrating cognitive health initiatives into existing health promotion frameworks, we can more effectively address the multifaceted contributors to mortality risk, particularly among individuals already practicing healthy lifestyle behaviors.

## CONFLICT OF INTEREST STATEMENT

All authors report no conflicts of interest. Author disclosures are available in the Supporting Information.

## CONSENT STATEMENT

All human subjects provided informed consent.

## Supporting information



Supporting Information

Supporting Information

Supporting Information

Supporting Information
