# Supplementary figures and images for: Association between healthy lifestyle and cognitive decline, all‐cause mortality, and mortality from cardiovascular and cerebrovascular diseases: a 10‐year population‐based prospective cohort study
Source: Alzheimers Dement. 2025 Mar 20;21(3):e70021. doi: 10.1002/alz.70021 (PMC11923566; doi:10.1002/alz.70021)

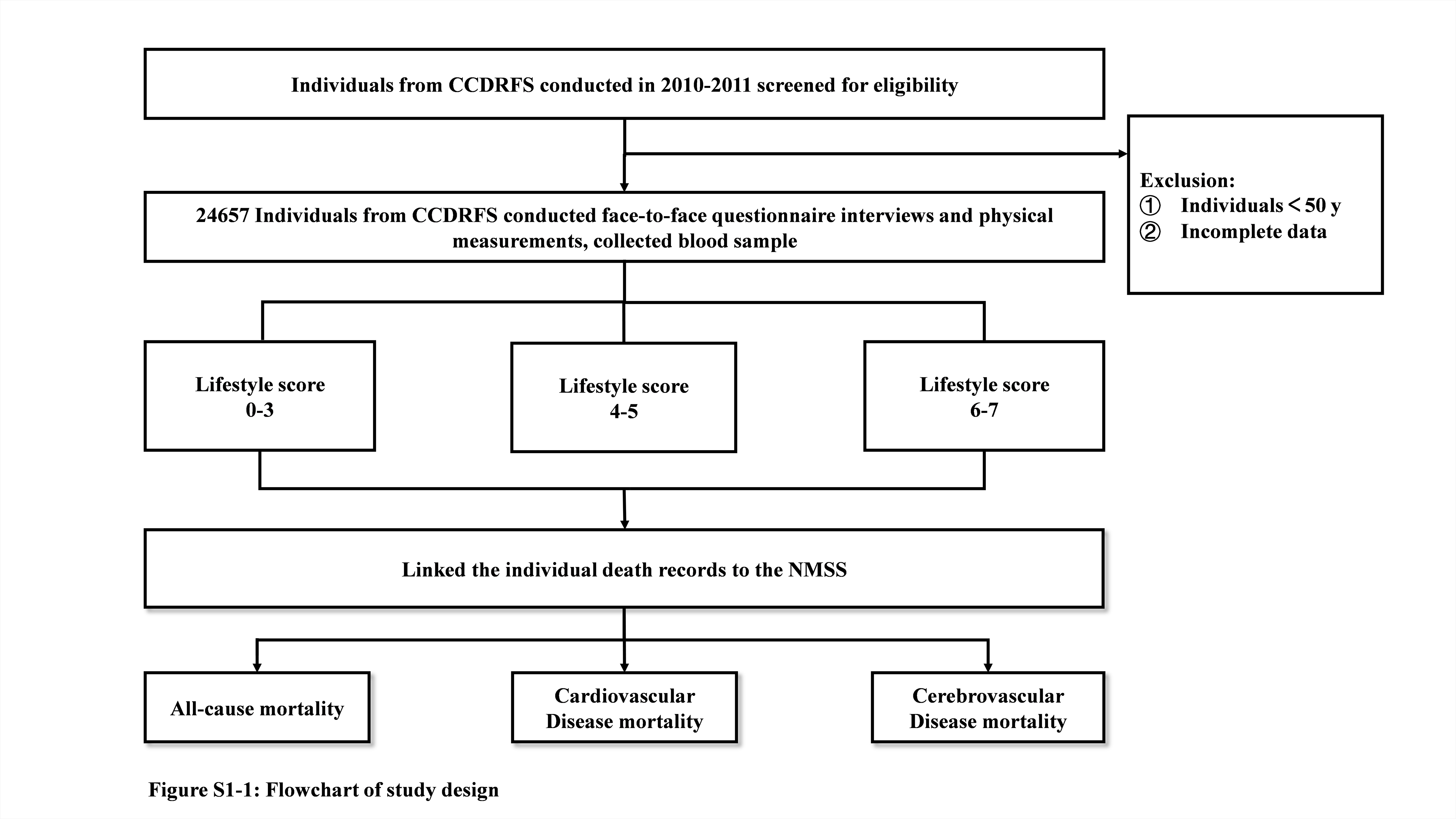

Supplement: Supplementary file 1 — Supporting Information [file ALZ-21-e70021-s001.tif]

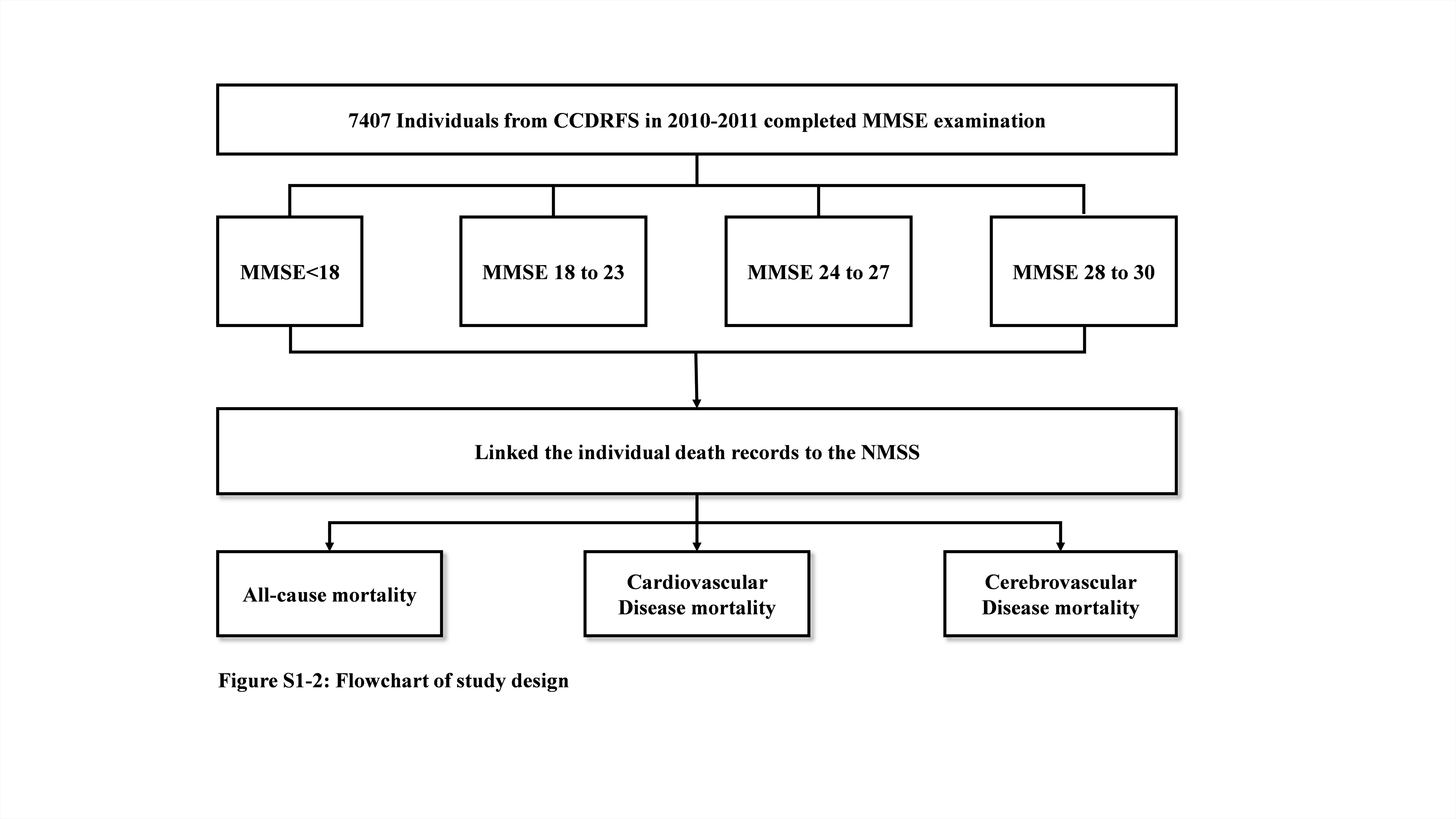

Supplement: Supplementary file 2 — Supporting Information [file ALZ-21-e70021-s003.tif]
