# Supplementary material for: Association between healthy lifestyle and cognitive decline, all‐cause mortality, and mortality from cardiovascular and cerebrovascular diseases: a 10‐year population‐based prospective cohort study
Source: Alzheimers Dement. 2025 Mar 20;21(3):e70021. doi: 10.1002/alz.70021 (PMC11923566; doi:10.1002/alz.70021)
Supplement: Supplementary file 3 — Supporting Information [file ALZ-21-e70021-s004.docx]

[**Online Supplemental Material**](#_Toc183617210)

[**Figure S1: Flowchart of study design 2**](#_Toc183617211)

[**Table S1. English and Chinese version of the questionnaire on life style factors in the China Chronic Disease and Risk Factor Surveillance 2010 and 2011 3**](#_Toc183617212)

[**Table S2. HR (95% CI) of cardiovascular mortality based on individual and combined lifestyle factors 6**](#_Toc183617213)

[**Table S3. HR (95% CI) of cerebrovascular mortality based on individual and combined lifestyle factors 7**](#_Toc183617214)

[**Table S4. Age-stratified associations between baseline MMSE scores and risk for all-cause, cardiovascular, and cerebrovascular disease mortality 8**](#_Toc183617215)

[**Table S5. Gender-stratified analysis of baseline MMSE scores and mortality risk for all-cause, cardiovascular, and cerebrovascular diseases 9**](#_Toc183617216)

[**Table S6. Association of baseline MMSE scores with cardiovascular disease mortality: stratification by lifestyle factors 10**](#_Toc183617217)

[**Table S7. Association of baseline MMSE scores with cerebrovascular disease mortality: stratification by lifestyle factors 11**](#_Toc183617218)

**Figure S1-1: Flowchart of study design**

**Figure S1-2: Flowchart of study design**

Note: CCDRFS represents China Chronic Disease and Risk Factor Surveillance; NMSS represents National Mortality Surveillance System; MMSE represents Mini-Mental State Examination.

**Table S1. English and Chinese version of the questionnaire on life style factors in the China Chronic Disease and Risk Factor Surveillance 2010 and 2011**

| **Code** | **English version** | | **Chinese version** | |
| --- | --- | --- | --- | --- |
|  | **Question** | **Answer** | **问题** | **选项** |
| B1 | Do you currently smoke daily, occasionally, or not at all? | 1.Yes, daily.  2.Yes, but not daily.  3.No, I do not smoke | 您现在吸烟吗，每天吸、不是每天吸、还是不吸？ | 1.是的，每天吸  2.是的，但不是每天吸  3.不吸 |
| B14 | Have you smoked in the past? | 1. Yes, daily  2. Yes, but not daily  3. No, I have never smoked  99. Do not know | 您过去是否吸烟？ | 1.是，每天吸  2.是，但不是每天吸  3.不吸  99.不知道 |
| B15 | How long has it been since you stopped smoking? | a.□□□ years  b.□□□ months  c.□□□ weeks  d.□□□ days | 您停止吸烟多长时间了？ | a.□□□年  b.□□□月  c.□□□周  d.□□□日 |
| C1 | Have you consumed alcohol in the past 12 months? | 1.Yes, within the past 30 days.  2.Yes, but more than 30 days ago.  3.No, I have not consumed alcohol. | 过去 12 个月里，您喝过酒吗？ | 1.喝过，在过去 30 天内  2.喝过，在 30 天前  3.没喝过 |
| E3 | How much time, in total, do you spend on high-intensity activities related to work, farming, or household chores in a typical day? | □□hours□□minutes | 在您的工作、农活及家务活动中，通常一天内累计有多长时间进行上述高强度活动？ | □□小时□□分钟 |
| E6 | How much time, in total, do you spend on moderate-intensity activities related to work, farming, or household chores in a typical day? | □□hours□□minutes | 在您的工作、农活及家务活动中，通常一天内累计有多长时间进行上述中等强度活动？ | □□小时□□分钟 |
| D5 | How much grain-based food (e.g., rice, wheat, mixed grains) do you consume in a day? (Recorded as raw weight) | □□.□liang | 米、面、杂粮 等粮谷类食物(按生重记录) | □□.□两 |
| D6 | How much starchy food (e.g., potatoes, taro, sweet potatoes) do you consume in a day? | □□.□liang | 薯类（土豆/ 芋头/红薯） | □□.□两 |
| D7 | How much pork do you consume in a day? (Recorded as raw weight) | □□.□liang | 猪肉 ( 按生重记录) | □□.□两 |
| D8 | How much red meat (e.g., beef, mutton) do you consume in a day? (Recorded as raw weight) | □□.□liang | 牛、羊等畜肉 ( 按生重记录) | □□.□两 |
| D9 | How much poultry (e.g., chicken, duck, goose) do you consume in a day? | □□.□liang | 鸡鸭鹅等禽肉 | □□.□两 |
| D10 | How much seafood (e.g., fish, shrimp) do you consume in a day? (Recorded as raw weight) | □□.□liang | 水产品 (鱼虾类，按生重计) | □□.□两 |
| D11 | How much fresh vegetables do you consume in a day? | □□.□liang | 新鲜蔬菜 | □□.□两 |
| D12 | How much fresh fruit do you consume in a day? | □□.□liang | 新鲜水果 | □□.□两 |
| D14 | How many eggs (measured as chicken eggs) do you consume in a day? | □□.□liang | 蛋类(以鸡蛋计) | □□.□两 |
| D15 | How much dairy (measured as fresh milk) do you consume in a day? | □□.□liang | 奶制品(以鲜奶计) | □□.□两 |
| E19 | How much time, in total, do you sleep in a typical day? | □□hours□□minutes | 通常一天内，您睡觉累计有多少时间？ | □□小时□□分钟 |
| E16 | How much time, in total, do you spend sitting, reclining, or lying down in a typical day? (This includes sedentary activities such as working, studying, reading, watching television, using the computer, or resting, but excludes sleep time.) | □□hours□□minutes | 通常一天内，您累计有多少时间坐着、靠着或躺着？（包括坐着工作、学习、阅读、看电视、用 电脑、休息等所有静态行为的时间，但不包括睡觉时间） | □□小时□□分钟 |
| H2 | Who do you currently live with? | 1.Family members.  2.Living alone.  3.In a nursing home or assisted living facility. | 您目前是跟谁生活在一起？ | 1家人  2独居  3敬老院/养老院 |

Note: A healthy lifestyle score is defined by assigning 1 point to each low-risk behavior, with all other behaviors receiving a score of 0. Non-smoking or having quit smoking for at least three years is considered low-risk, where current smokers are identified as those responding with "1" or "2" to B1, former smokers as those reporting a cessation period of three or more years in B15, and non-smokers as those responding with "3" to both B1 and B14. Non-drinking is defined as never consuming alcohol, indicated by a response of "3" to C1. Healthy physical activity is determined as engaging in at least 150 minutes of moderate-intensity activity or 75 minutes of high-intensity activity per week, based on responses to E3 and E6. A healthy diet requires meeting or exceeding five out of ten specified dietary categories, including D5-D12 and D14-D15. Adequate sleep is defined as sleeping 7 to 8 hours per day. Total sedentary time is considered low-risk if it is less than 4 hours per day, which includes time spent on activities such as watching television, using a computer, reading, playing video games, or browsing the internet during leisure time, derived from responses to E16. Lastly, social engagement is deemed healthy for individuals living with family (response "1" to H2) or in a nursing or retirement home (response "3" to H2), while those living alone (response "2" to H2) receive a score of 0.

**Table S2. HR (95% CI) of cardiovascular mortality based on individual and combined lifestyle factors**

|  |  | **HR(95% *CI*)** | | |
| --- | --- | --- | --- | --- |
|  | **No. of cases/100000 person-years** | **Model 1** | **Model 2** | **Model 3** |
| Smoking |  |  |  |  |
| Previous/Never | 169.34 | 1 (Reference) | 1 (Reference) | 1 (Reference) |
| Current | 225.38 | 1.17(0.94-1.45) | 1.14(0.92-1.42) | 1.16(0.93-1.43) |
| Alcohol consumption |  |  |  |  |
| Never | 197.88 | 1 (Reference) | 1 (Reference) | 1 (Reference) |
| Previous/Current | 173.73 | 0.90(0.74-1.10) | 0.92(0.76-1.13) | 0.95(0.78-1.16) |
| Physical activity |  |  |  |  |
| Adequate | 166.43 | 1 (Reference) | 1 (Reference) | 1 (Reference) |
| Inadequate | 320.17 | 1.32(1.08-1.60)^*^ | 1.31(1.08-1.60)^*^ | 1.30(1.07-1.59)^*^ |
| Healthy diet |  |  |  |  |
| Yes | 186.91 | 1 (Reference) | 1 (Reference) | 1 (Reference) |
| No | 318.13 | 1.50(0.96-2.34) | 1.42(0.90-2.22) | 1.34(0.85-2.11) |
| Sleep duration, h/day |  |  |  |  |
| 7-8 | 170.87 | 1 (Reference) | 1 (Reference) | 1 (Reference) |
| ≤6 | 210.13 | 1.03(0.84-1.25) | 0.99(0.82-1.21) | 0.97(0.80-1.19) |
| ≥9 | 217.16 | 1.09(0.88-1.36) | 1.07(0.85-1.33) | 1.05(0.84-1.31) |
| Sedentary Time, h/day |  |  |  |  |
| ＜4 | 187.81 | 1 (Reference) | 1 (Reference) | 1 (Reference) |
| ≥4 | 200.03 | 1.12(0.90-1.41) | 1.24(0.98-1.57) | 1.23(0.97-1.56) |
| Living alone |  |  |  |  |
| No | 177.63 | 1 (Reference) | 1 (Reference) | 1 (Reference) |
| Yes | 355.34 | 1.09(0.85-1.42) | 0.80(0.60-1.07) | 0.81(0.60-1.08) |
| Overall lifestyle score |  |  |  |  |
| 6-7 | 153.62 | 1 (Reference) | 1 (Reference) | 1 (Reference) |
| 4-5 | 228.68 | 1.23(1.03-1.48)^†^ | 1.18(0.98-1.41) | 1.20(1.00-1.44) |
| 0-3 | 272.14 | 1.25(0.86-1.82) | 1.14(0.78-1.66) | 1.14(0.78-1.67) |

Note: Model 1 adjusted for age, sex; Model 2 adjusted for age, sex, education level, marital status, ethnicity, residence, BMI, income, occupation; Model 3 adjusted for Age, sex, education level, marital status, ethnicity, residence, BMI, income, occupation, hypertension, diabetes mellitus, hyperlipidemia, myocardial infarction, cerebrovascular disease, COPD, cancer, traumatic brain injury, depression. HR represents Hazard Ratio. *P<0.001. †P<0.05.

**Table S3.** **HR (95% CI) of cerebrovascular mortality based on individual and combined lifestyle factors**

|  |  | **HR (95% *CI*)** | | |
| --- | --- | --- | --- | --- |
|  | **No. of cases/100000 person-years** | **Model 1** | **Model 2** | **Model 3** |
| Smoking |  |  |  |  |
| Previous/Never | 232.22 | 1 (Reference) | 1 (Reference) | 1 (Reference) |
| Current | 296.34 | 1.05(0.87-1.26) | 1.00(0.83-1.21) | 1.02(0.85-1.23) |
| Alcohol consumption |  |  |  |  |
| Never | 263.14 | 1 (Reference) | 1 (Reference) | 1 (Reference) |
| Previous/Current | 240.70 | 0.91(0.77-1.08) | 0.95(0.80-1.12) | 0.95(0.80-1.13) |
| Physical activity |  |  |  |  |
| Adequate | 232.09 | 1 (Reference) | 1 (Reference) | 1 (Reference) |
| Inadequate | 386.97 | 1.18(0.99-1.40) | 1.14(0.96-1.36) | 1.14(0.95-1.36) |
| Healthy diet |  |  |  |  |
| Yes | 250.65 | 1 (Reference) | 1 (Reference) | 1 (Reference) |
| No | 477.20 | 1.69(1.18-2.44)^*^ | 1.43(0.99-2.07) | 1.36(0.94-1.97) |
| Sleep duration, h/day |  |  |  |  |
| 7-8 | 213.42 | 1 (Reference) | 1 (Reference) | 1 (Reference) |
| ≤6 | 285.09 | 1.13(0.95-1.34) | 1.09(0.92-1.30) | 1.06(0.89-1.27) |
| ≥9 | 338.84 | 1.37(1.14-1.64)^*^ | 1.27(1.06-1.52)^†^ | 1.23(1.03-1.48)^†^ |
| Sedentary Time, h/day |  |  |  |  |
| ＜4 | 275.65 | 1 (Reference) | 1 (Reference) | 1 (Reference) |
| ≥4 | 152.71 | 0.57(0.44-0.73)^*^ | 0.72(0.56-0.93)^†^ | 0.72(0.56-0.93)^†^ |
| Living alone |  |  |  |  |
| No | 232.58 | 1 (Reference) | 1 (Reference) | 1 (Reference) |
| Yes | 563.46 | 1.42(1.15-1.75)^*^ | 1.12(0.88-1.42) | 1.13(0.89-1.44) |
| Overall lifestyle score |  |  |  |  |
| 6-7 | 209.77 | 1 (Reference) | 1 (Reference) | 1 (Reference) |
| 4-5 | 308.00 | 1.20(1.03-1.41)^†^ | 1.16(0.99-1.36) | 1.17(1.00-1.38)^†^ |
| 0-3 | 329.87 | 1.10(0.79-1.55) | 1.02(0.72-1.43) | 1.00(0.71-1.41) |

Note: Model 1 adjusted for age, sex; Model 2 adjusted for age, sex, education level, marital status, ethnicity, residence, BMI, income, occupation; Model 3 adjusted for Age, sex, education level, marital status, ethnicity, residence, BMI, income, occupation, hypertension, diabetes mellitus, hyperlipidemia, myocardial infarction, cerebrovascular disease, COPD, cancer, traumatic brain injury, depression. HR represents Hazard Ratio. ^*^*P*<0.001. ^†^*P*<0.05.

**Table S4. Age-stratified associations between baseline MMSE scores and risk for all-cause, cardiovascular, and cerebrovascular disease mortality**

|  | Age＜65 | | Age ≥65 | |  |
| --- | --- | --- | --- | --- | --- |
|  | No. of cases/100000 person-years | HR (95%*CI*) | No. of cases/100000 person-years | HR (95%*CI*) | *P* for interaction |
| **All-cause mortality** |  |  |  |  | <0.0001 |
| MMSE<18 | 883.39 | 1.62(1.10-2.39)^†^ | 3079.16 | 1.47(1.15-1.88)^†^ |  |
| MMSE 18 to 23 | 797.96 | 1.52(1.16-2.00)^†^ | 2578.77 | 1.21(0.96-1.52) |  |
| MMSE 24 to 27 | 745.06 | 1.30(1.02-1.66)^†^ | 2474.81 | 1.08(0.86-1.35) |  |
| MMSE 28 to 30 | 592.27 | 1(Reference) | 2120.97 | 1(Reference) |  |
| **Cardiovascular Disease mortality** | |  |  |  | <0.0001 |
| MMSE<18 | 126.20 | 1.76(0.62-5.01) | 615.83 | 0.96(0.58-1.60) |  |
| MMSE 18 to 23 | 109.19 | 1.61(0.77-3.38) | 609.06 | 0.95(0.61-1.50) |  |
| MMSE 24 to 27 | 76.42 | 0.95(0.46-1.98) | 475.39 | 0.72(0.45-1.15) |  |
| MMSE 28 to 30 | 73.07 | 1(Reference) | 565.59 | 1(Reference) |  |
| **Cerebrovascular Disease mortality** | |  |  |  | <0.0001 |
| MMSE<18 | 302.88 | 3.03(1.44-6.35)^†^ | 989.07 | 1.60(0.99-2.61) |  |
| MMSE 18 to 23 | 226.79 | 2.40(1.34-4.29)^†^ | 712.73 | 1.22(0.77-1.93) |  |
| MMSE 24 to 27 | 178.31 | 1.99(1.15-3.44)^†^ | 601.23 | 1.05(0.66-1.67) |  |
| MMSE 28 to 30 | 88.46 | 1(Reference) | 452.47 | 1(Reference) |  |

Note: Model 1 adjusted for age, sex; Model 2 adjusted for age, sex, education level, marital status, ethnicity, residence, BMI, income, occupation; Model 3 adjusted for Age, sex, education level, marital status, ethnicity, residence, BMI, income, occupation, hypertension, diabetes mellitus, hyperlipidemia, myocardial infarction, cerebrovascular disease, COPD, cancer, traumatic brain injury, depression. MMSE represents Mini-Mental State Examination. HR represents Hazard Ratio. *P<0.001. †P<0.05.

**Table S5. Gender-stratified analysis of baseline MMSE scores and mortality risk for all-cause, cardiovascular, and cerebrovascular diseases**

|  | Male | | Female | |  |
| --- | --- | --- | --- | --- | --- |
|  | No. of cases/100000 person-years | HR (95%*CI*) | No. of cases/100000 person-years | HR (95%*CI*) | *P* for interaction |
| **All-cause mortality** | |  |  |  | <0.0001 |
| MMSE<18 | 2858.98 | 1.37(1.03-1.83)^†^ | 1845.25 | 1.57(1.15-2.14)^†^ |  |
| MMSE 18 to 23 | 2130.62 | 1.31(1.04-1.65)^†^ | 1173.06 | 1.36(1.03-1.80)^†^ |  |
| MMSE 24 to 27 | 1752.39 | 1.22(0.99-1.49) | 882.25 | 1.19(0.90-1.58) |  |
| MMSE 28 to 30 | 1276.11 | 1(Reference) | 562.29 | 1(Reference) |  |
| **Cardiovascular** **Disease mortality** | | |  |  | <0.01 |
| MMSE<18 | 506.65 | 0.74(0.38-1.45) | 366.00 | 1.37(0.69-2.71) |  |
| MMSE 18 to 23 | 306.11 | 0.83(0.48-1.46) | 277.83 | 1.60(0.89-2.91) |  |
| MMSE 24 to 27 | 216.69 | 0.61(0.36-1.05) | 187.89 | 1.20(0.64-2.24) |  |
| MMSE 28 to 30 | 247.96 | 1(Reference) | 108.83 | 1(Reference) |  |
| **Cerebrovascular Disease mortality** | | |  |  | <0.0001 |
| MMSE<18 | 868.55 | 1.80(1.02-3.19)^†^ | 625.25 | 2.02(1.08-3.78)^†^ |  |
| MMSE 18 to 23 | 630.18 | 1.79(1.12-2.88)^†^ | 308.70 | 1.52(0.85-2.72) |  |
| MMSE 24 to 27 | 452.23 | 1.56(1.00-2.44)^†^ | 187.89 | 1.28(0.71-2.32) |  |
| MMSE 28 to 30 | 223.77 | 1(Reference) | 108.83 | 1(Reference) |  |

Note: Model 1 adjusted for age, sex; Model 2 adjusted for age, sex, education level, marital status, ethnicity, residence, BMI, income, occupation; Model 3 adjusted for Age, sex, education level, marital status, ethnicity, residence, BMI, income, occupation, hypertension, diabetes mellitus, hyperlipidemia, myocardial infarction, cerebrovascular disease, COPD, cancer, traumatic brain injury, depression. HR represents Hazard Ratio. MMSE represents Mini-Mental State Examination. ^*^*P*<0.001. ^†^*P*<0.05.

**Table S6. Association of baseline MMSE scores with cardiovascular disease mortality: stratification by lifestyle factors**

Note: Model 1 adjusted for age, sex; Model 2 adjusted for age, sex, education level, marital status, ethnicity, residence, BMI, income, occupation; Model 3 adjusted for Age, sex, education level, marital status, ethnicity, residence, BMI, income, occupation, hypertension, diabetes mellitus, hyperlipidemia, myocardial infarction, cerebrovascular disease, COPD, cancer, traumatic brain injury, depression. HR represents Hazard Ratio. MMSE represents Mini-Mental State Examination. ^*^*P*<0.001. ^†^*P*<0.05.

|  | HR (95%*CI*) | | | | | |
| --- | --- | --- | --- | --- | --- | --- |
|  | Model 1 | | Model 2 | | Model 3 | |
| **Cardiovascular Disease mortality** | | | | | |  |
| **Lifestyle score 0 to 3** |  |  |  |  |  |  |
| MMSE<18 | 2.55(0.47~13.92) | | 0.72(0.11~4.52) | | 13.74(0.29~642.63) | |
| MMSE 18 to 23 | 1.37(0.25~7.47) | | 0.75(0.13~4.41) | | 0.99(0.06~16.75) | |
| MMSE 24 to 27 | 0.74(0.14~4.06) | | 0.44(0.08~2.58) | | 0.37(0.03~4.67) | |
| MMSE 28 to 30 | 1 (Reference) | | 1 (Reference) | | 1 (Reference) | |
| **Lifestyle score 4 to 5** |  |  |  |  |  |  |
| MMSE<18 | 2.20(1.22~3.97)^*^ | | 0.86(0.46~1.62) | | 0.71(0.35~1.41) | |
| MMSE 18 to 23 | 1.90(1.14~3.17)^†^ | | 1.10(0.65~1.88) | | 1.01(0.56~1.80) | |
| MMSE 24 to 27 | 0.96(0.54~1.70) | | 0.75(0.42~1.34) | | 0.67(0.37~1.22) | |
| MMSE 28 to 30 | 1 (Reference) | | 1 (Reference) | | 1 (Reference) | |
| **Lifestyle score 6 to 7** |  |  |  |  |  |  |
| MMSE<18 | 2.31(1.29~4.14)^*^ | | 1.41(0.76~2.59) | | 1.33(0.69~2.57) | |
| MMSE 18 to 23 | 1.65(1.00~2.75)^†^ | | 1.30(0.78~2.18) | | 1.15(0.67~1.99) | |
| MMSE 24 to 27 | 1.18(0.69~2.04) | | 1.04(0.60~1.79) | | 0.92(0.52~1.61) | |
| MMSE 28 to 30 | 1 (Reference) | | 1 (Reference) | | 1 (Reference) | |

**Table S7. Association of baseline MMSE scores with cerebrovascular disease mortality: stratification by lifestyle factors**

|  | HR (95%*CI*) | | | | | |
| --- | --- | --- | --- | --- | --- | --- |
|  | Model 1 | | Model 2 | | Model 3 | |
| **Cerebrovascular Disease mortality** | | | | | | |
| **Lifestyle score 0 to 3** |  |  |  |  |  |  |
| MMSE<18 | 3.30(0.79~13.83) | | 1.94(0.40~9.45) | | 0.17(0.02~1.28) | |
| MMSE 18 to 23 | 1.18(0.23~6.07) | | 0.86(0.16~4.59) | | 0.16(0.02~1.16) | |
| MMSE 24 to 27 | 1.80(0.55~5.90) | | 1.45(0.42~4.93) | | 0.26(0.05~1.42) | |
| MMSE 28 to 30 | 1 (Reference) | | 1 (Reference) | | 1 (Reference) | |
| **Lifestyle score 4 to 5** |  |  |  |  |  |  |
| MMSE<18 | 3.36(2.07~5.46)^*^ | | 1.82(1.08~3.06)^†^ | | 1.21(0.69~2.11) | |
| MMSE 18 to 23 | 2.17(1.37~3.44)^*^ | | 1.53(0.95~2.46) | | 1.12(0.67~1.84) | |
| MMSE 24 to 27 | 1.62(1.02~2.56)^†^ | | 1.37(0.87~2.18) | | 1.11(0.69~1.79) | |
| MMSE 28 to 30 | 1 (Reference) | | 1 (Reference) | | 1 (Reference) | |
| **Lifestyle score 6 to 7** |  |  |  |  |  |  |
| MMSE<18 | 5.61(3.07~10.26)^*^ | | 4.04(2.16~7.54)^*^ | | 4.48(2.29~8.80)^*^ | |
| MMSE 18 to 23 | 3.84(2.20~6.71)^*^ | | 3.29(1.87~5.79)^*^ | | 3.41(1.86~6.25)^*^ | |
| MMSE 24 to 27 | 2.60(1.45~4.69)^*^ | | 2.36(1.31~4.27)^*^ | | 2.36(1.29~4.33)^*^ | |
| MMSE 28 to 30 | 1 (Reference) | | 1 (Reference) | | 1 (Reference) | |

Note: Model 1 adjusted for age, sex; Model 2 adjusted for age, sex, education level, marital status, ethnicity, residence, BMI, income, occupation; Model 3 adjusted for Age, sex, education level, marital status, ethnicity, residence, BMI, income, occupation, hypertension, diabetes mellitus, hyperlipidemia, myocardial infarction, cerebrovascular disease, COPD, cancer, traumatic brain injury, depression. HR represents Hazard Ratio. MMSE represents Mini-Mental State Examination. ^*^*P*<0.001. ^†^*P*<0.05.
